# Study on the Mechanism of Fisetin Exerting Anti‐Liver Cancer Effects by Regulating Neutrophil Extracellular Traps

**DOI:** 10.1002/fsn3.70309

**Published:** 2025-06-18

**Authors:** Jiahui Gao, Yujia Song, Zanxiang Luo, Zejie Su, Chengshi Fu, Anran Gao, Jingxiu Zhao, Lie Liu, Xiangyun Teng, Jianhua Xu

**Affiliations:** ^1^ Department of Laboratory Medicine Shunde Hospital of Guangzhou University of Chinese Medicine Foshan China; ^2^ Department of Clinical Laboratory Maoming Hospital of Guangzhou University of Chinese Medicine Maoming China; ^3^ Translational Research Center for Traditional Chinese Medicine Maoming Hospital of Guangzhou University of Chinese Medicine Maoming China

**Keywords:** AKT, fisetin, liver cancer, neutrophil extracellular traps, ROS

## Abstract

Liver cancer (LC) is among the most prevalent malignant tumors in the digestive tract. The high incidence rate, high metastasis and recurrence rate, poor prognosis, and heterogeneity of liver cancer seriously threaten human health. It is very necessary to search for new drugs and new targets against liver cancer. Natural drugs have been proved to have good anti‐tumor effects. Fisetin, a dietary flavonoid often found in fruits and vegetables, has multiple pharmacological functions such as anti‐inflammation, anti‐oxidation, immune regulation, and anti‐tumor. Firstly, we found in vitro that fisetin could inhibit the proliferation, migration, and invasion of liver cancer cells. Bioinformatics analysis speculated that its anti‐liver cancer mechanism might be related to the formation of neutrophil extracellular traps (NETs). Then, neutrophils were extracted from healthy volunteers, and it was found that fisetin might inhibit the formation of NETs through the AKT/ROS axis. Then, liver cancer cells were cultured in the medium containing NETs. We found that fisetin could weaken the proliferation, migration, and invasion of liver cancer cells induced by NETs. Finally, we found that fisetin could inhibit tumor growth in C57BL/6 mice. Fisetin could inhibit the recruitment of neutrophils and the level of NETs in tumor tissues. In conclusion, we found that fisetin could be used as a new NETs inhibitor and further clarified the anti‐liver cancer effect of fisetin.

## Graphical abstract

In this study, we aim to explore the mechanism of the anti‐liver cancer effect of fisetin through in vivo and in vitro experiments. We found that fisetin can inhibit the formation of NETs. The mechanism may be related to the inhibition of p‐AKT protein and the reduction of ROS production in neutrophils. Meanwhile, we found in vivo that fisetin could inhibit the recruitment of neutrophils and the level of NETs in tumor tissues, thereby exerting an anti‐liver effect. We proposed a new NETs inhibitor and provided a new target for the treatment of liver cancer.

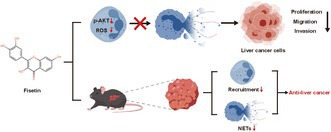

## 1 Introduction

Liver cancer (LC) is one of the most common malignant tumors. Its global cancer incidence ranks sixth, and its mortality rate ranks third. In China, its cancer incidence ranks fourth, and its mortality rate ranks second (Sung et al. 2021). Common incentives for liver cancer include hepatitis virus infection, metabolic disorder, alcohol, immune dysfunction, and drug‐induced liver injury (Reig et al. 2018). Liver transplantation, tumor resection, molecular targeted therapy, interventional therapy, and drug chemotherapy are all currently commonly used clinical liver cancer treatment methods. However, due to the clinical characteristics of liver cancer, such as no obvious symptoms in the early stage, rapid disease progression, high metastasis, and insensitivity to chemotherapy, the surgical efficacy of most patients is not good, and they are prone to various complications. Moreover, chemotherapy drugs are prone to drug resistance during use, and the patient's quality of life has not been significantly improved (Anwanwan et al. 2020). Therefore, it is extremely essential to seek new drugs and new targets for anti‐liver cancer.

Many studies have found that some natural components extracted from plants have obvious advantages in the process of tumor treatment, such as low toxicity, small side effects, multiple targets of action, and low tendency to develop drug resistance, which can provide new options for inhibiting the occurrence and development of tumors (Zhang et al. 2025; Zhu et al. 2023). Fisetin is a common dietary flavonoid, widely present in fruits and vegetables such as apples, strawberries, onions, and cucumbers. Fisetin is widely used in disease treatment (Kashyap et al. 2019). Studies have shown that fisetin can exert anti‐tumor effects in vitro and in vivo through multiple targets and pathways, including signal pathways such as mTOR (Roy et al. 2021), Wnt/β‐catenin (Fu et al. 2025), NF‐κB, and AMPK (Afroze et al. 2022), but its anti‐liver cancer mechanism remains to be clarified.

The tumor microenvironment (TME) is the “soil” for the growth of tumor cells and is composed of various cellular and non‐cellular factors. Neutrophils are heterogeneous white blood cells present in the TME. Under the stimulation of cytokines secreted by various tumors, they will release their own nucleic acids, proteins, and other substances. A reticular structure with DNA as the framework and embedded with granular proteins such as neutrophil elastase (NE) and myeloperoxidase (MPO) is formed, which is called neutrophil extracellular traps (NETs) (Herre et al. 2023), and this process is called NETosis. Studies have shown that the levels of NETs markers in the serum and tumor tissues of patients with liver cancer are higher than those of healthy people (Kajioka et al. 2021). NETs promote the growth and metastasis of hepatocellular carcinoma (HCC), mainly manifested as increased angiogenesis, increased cell migration related to epithelial–mesenchymal transition (EMT) (Jiang et al. 2022), increased extracellular matrix (ECM) degradation induced by matrix metalloproteinases (MMPs), and increased cell capture mediated by NETs (Yang et al. 2020). Therefore, inhibiting the formation of NETs may be a potential target for the treatment of liver cancer.

We investigated the anti‐liver cancer effect of fisetin both in vivo and in vitro. The mechanism might be associated with the inhibition of neutrophil extracellular traps. We offer basic research support for the anti‐tumor effect of fisetin and present a new strategy for liver cancer treatment as well.

## 2 Materials and Methods

### 2.1 Reagents and Antibodies

DMEM medium (GIBICO, 11965092), fetal bovine serum (GIBICO, A5670801), RPMI 1670 medium (GIBICO, 12633020), PBS (GIBICO, 10010023), Fisetin (Herb‐substance, PCS0750), Crystal violet staining solution (Beyotime, C0121), matrigel (Beyotime, C0372) and DAPI (Beyotime, C1005), Transwell chambers (Corning, 353097), PMA (Aladdin, P408905), CitH3 antibody (Abcam, Ab5103), GAPDH (Proteintech, 10494‐1‐AP), MPO (Proteintech, 22225‐1‐AP), Ki67 (Proteintech, 27309‐1‐AP), Ly6G antibody (Biolegend, 127636), and CCK‐8 kit (C0038, Beyotime).

### 2.2 Cell Culture

Liver cancer cells, namely HepG2, Huh‐7, Hepa1‐6, and MHCC97H, were cultured in DMEM complete medium which contains 10% FBS and 1% antibiotics (penicillin and streptomycin). When the cell density reached 80%, the cells were subcultured for the next step of the experiment. Neutrophils were cultured in RPMI 1640 complete medium.

### 2.3 CCK‐8

Liver cancer cells were inoculated into 96‐well plates at a density of 5 × 10^3^ cells per well. Different drugs were used to treat the cells for either 24 h or 48 h. Subsequently, the medium with 10% CCK‐8 reagent was added. After continuous incubation for 1–2 h, the absorbance at 450 nm was measured by an enzyme marker.

Neutrophils were inoculated into 96‐well plates at 1 × 10^5^ cells/well. Ten percent CCK‐8 reagent was added directly at the indicated time after drug administration, and the absorbance was measured after 1–2 h of continued incubation.

### 2.4 Colony Formation Assay

Cells were inoculated in 12‐well plates at a density of 1 × 10^3^ cells/well. Then the medium was changed to complete medium containing different concentrations of fisetin after the cells were attached to the wall. After 10 days of continuous cultivation, the cells were fixed with 4% paraformaldehyde at room temperature for 15 min. Then stained with 0.1% crystal violet for 10 min, so as to count the number of cell communities.

### 2.5 Wound Healing Assay

Cells were inoculated into 6‐well plates at 6 × 10^5^ cells/well. When the cell density reached 90–100%, a uniform straight line was scratched in each well with a 10 μL tip. After washing away the cell debris with PBS, 2 mL of drug‐containing medium containing 2% FBS was added. Photographs were taken under a microscope (Olympus, Japan) at 0 h and 48 h. The area between the scratches was recorded using ImageJ software to calculate the percentage of migrated area.

### 2.6 Transwell Assay

To detect cell migration using the transwell assay, 800 μL of drug‐containing medium with 15% FBS was added to 24‐well plates. Cell suspensions were made using serum‐free medium. Two hundred microliters of cells were inoculated into the chambers at a density of 1 × 10^5^ cells/well. The chambers were removed after 48 h of incubation in the incubator. The cells in the chambers were then wiped with a moistened cotton swab. The cells were then fixed and stained. Finally, the cells were swabbed with PBS, examined under a microscope, and photographed in 3‐5 fields of view.

When using transwell assay to detect cell invasion, 60 μL of matrix gel mixed with serum‐free medium was evenly spread on the bottom of the chambers. It was incubated in the incubator for 2 h for coagulation, and then 100 μL of serum‐free medium was added for hydration. It was checked whether any liquid entered the lower chamber. If not, it could be used for cell inoculation. The remaining steps were the same as above.

### 2.7 Network Pharmacology

The related targets of fisetin were retrieved and duplicates were removed in the TCMSP database, Pharmmapper, and SwissTargetPrediction. The disease‐related targets were retrieved and duplicates were removed by using the keyword “liver cancer” in the Human Gene Database and the Online Mendelian Inheritance in Man Database. The information of disease targets and component targets was imported into Venny 2.1.0 to obtain their intersection, and the potential targets were initially obtained. The targets were imported into the STRING database to obtain the PPI network diagram and perform visual analysis through Cytoscape 3.10.0 software. Then, the 197 targets were imported into the online software Metascape for GO and KEGG enrichment analysis. The results obtained from the analysis were visualized through the online platform of Weishengxin.

### 2.8 Molecular Docking

The 3D structure of fisetin was obtained from the PubChem database. The structures of target proteins AKT (PDB ID: 3O96), NE (PDB ID: 5ABW), and MPO (PDB ID: 5FIW) were retrieved from the RCSB PDB database. The target protein structures were prepared by eliminating nonessential ligands and solvent molecules using PyMOL 2.5.7 software. Subsequent preprocessing—including hydrogen atom addition, charge assignment, and atom type definition—was conducted in AutoDockTools 1.5.7 software. Docked complexes were analyzed in PyMOL, with binding affinities evaluated based on computed binding energies.

### 2.9 Bioinformatics

The relationships between liver cancer, neutrophils, HIST3H3, and MPO were retrieved respectively in the Diff Exp, SCNA, and Gene modules of the TIMER public database. The immunohistochemical results of MPO in liver tissues of healthy people and tumor tissues of liver cancer patients were retrieved in the HPA database. See supplementary material for the web address.

### 2.10 Extraction of Neutrophils and Induction of NETs

Neutrophils were extracted by using polymorphprep separation solution (AS111468, Axis‐Shield). Collect EDTA anticoagulant whole blood from healthy volunteers, and add Polymorphprep cell separation liquid in a 15 mL centrifuge tube. Carefully layer the blood on the top of Polymorphprep at a 1:1 ratio. Centrifuge at 500 g for 30 min, and then aspirate the middle white annular layer. Centrifuge at 400 g for 10 min, discard the supernatant, wash with PBS, and then add red blood cell lysis buffer. Centrifuge at 400 g for 10 min and discard the supernatant. Then, it was washed with PBS once again to obtain neutrophils.

The isolated neutrophils were resuspended in medium and seeded in 96‐well plates at a density of 1 × 10^5^ cells/well in 100 μL medium. The cells were then treated with 25 nM PMA and fisetin for 4 h. After incubation, the supernatant was carefully removed. The cells were rinsed with PBS. Centrifuge at 1500 rpm for 10 min at 4°C to separate NETs, which were subsequently collected from the supernatant for further analysis.

### 2.11 cfDNA Analysis

The cfDNA was detected by the dsDNA detection kit (P7581, Thermo Fisher Scientific). Firstly, prepare the working solution at a ratio of DMSO:dsDNA Reagent = 1:199. Take 50 μL of each sample and add it to 50 μL of TE Buffer, then add 100 μL of the working solution. Incubate in the dark for 2–5 min. Fluorescence intensity was measured using a multi‐functional microplate reader. The EX = 480 nm and the EW = 520 nm. The standard curve was then plotted, and the cf DNA content was calculated.

### 2.12 Immunofluorescence

Neutrophils were inoculated at a density of 5 × 10^5^ cells/well onto the polylysine‐coated coverslips. Following 4 h of treatment, the cells were blocked for 30 min with 5% BSA and fixed for 20 min with 4% paraformaldehyde. The fluorescent secondary antibody was incubated for 1 h at room temperature after the primary antibody had been treated for the entire night at 4°C. The cell nuclei were stained using DAPI staining solution. Following a PBS rinse, the cells were examined and captured on camera using a fluorescent microscope.

### 2.13 ROS Analysis

Neutrophils were seeded in 6‐well plates at a density of 5 × 10^5^ cells/well. 3 h after treatment, a positive control was added to the cells and incubated at 37°C for 1 h in the dark to increase reactive oxygen species levels. Cells were collected by centrifugation, diluted H2DCFDA probe was added, and incubated at 37°C for 30 min in the dark. Fluorescence intensity was observed under a microscope. Mean fluorescence intensity (MFI) of cells was determined by flow cytometry.

### 2.14 Animal Experiments

Male C57BL/6J mice (4–6 weeks, 18–20 g) were sourced from the Guangdong Medical Laboratory Animal Center and housed under specific pathogen‐free (SPF) conditions. To develop the subcutaneous liver cancer model, 2 × 10^6^ Hepa1‐6 cells were injected into the left scapular region of each mouse. When tumor sizes reached 100–200 mm^3^, the mice were randomly allocated into experimental groups. The fisetin‐treated group was administered daily intraperitoneal injections of fisetin at a dosage of 80 mg/kg/day for 24 days, whereas the control group received an equal volume of vehicle. Every 4 days, calipers were used to record tumor dimensions, and the formula: Volume = Length × Width^2^/2 was utilized to calculate tumor volume. 24 h after the final treatment, the mice were humanely euthanized, and tumors were harvested, weighed, and photographed for documentation.

### 2.15 Western Blotting

RIPA was used to extract protein from tissues or cells. SDS‐PAGE was used to separate proteins on 10%–12% gels. Proteins were subsequently put onto PVDF. After blocking the membranes, the membranes underwent three TBST washes. Incubate the primary antibody for an entire night at 4°C and the secondary antibody for 1 h at room temperature. A reagent called enhanced chemiluminescence (ECL) is used to view protein bands. ImageJ software was used to quantify the bands' intensity.

### 2.16 Immunohistochemical

The tissue samples were placed in the fixative solution for 48 h and then subjected to dehydration and embedding. After wax removal, slices were rehydrated in 10 mmol/L citrate buffer. They were then treated with 3% H_2_O_2_/MeOH. To prevent nonspecific binding, slices were treated with 5% BSA blocking solution. Incubate the primary antibody for an entire night at 4°C and the secondary antibody for 1 h at room temperature. Sections were washed with PBS and stained with the DAB substrate kit. Then, they were photographed under a microscope, and statistically analyzed for the percentage of positive sections using ImageJ software.

### 2.17 Statistical Analysis

Statistical analysis and data visualization in this study were performed using SPSS 27.0 and GraphPad Prism 9.4.0 software. For comparisons between two groups, an independent sample *t*‐test was applied if the data exhibited a normal distribution and homogeneity of variance. In cases involving comparisons among multiple groups, one‐way analysis of variance (ANOVA) was employed, provided the data met the assumptions of normality and equal variance. When the data deviated from a normal distribution or displayed heterogeneity of variance, non‐parametric tests were utilized. A paired samples t‐test was employed when the paired data followed a normal distribution. P value less than 0.05 was considered statistically significant.

## 3 Results

### 3.1 Fisetin Inhibits the Proliferation of Liver Cancer Cells In Vitro

**FIGURE 1:**
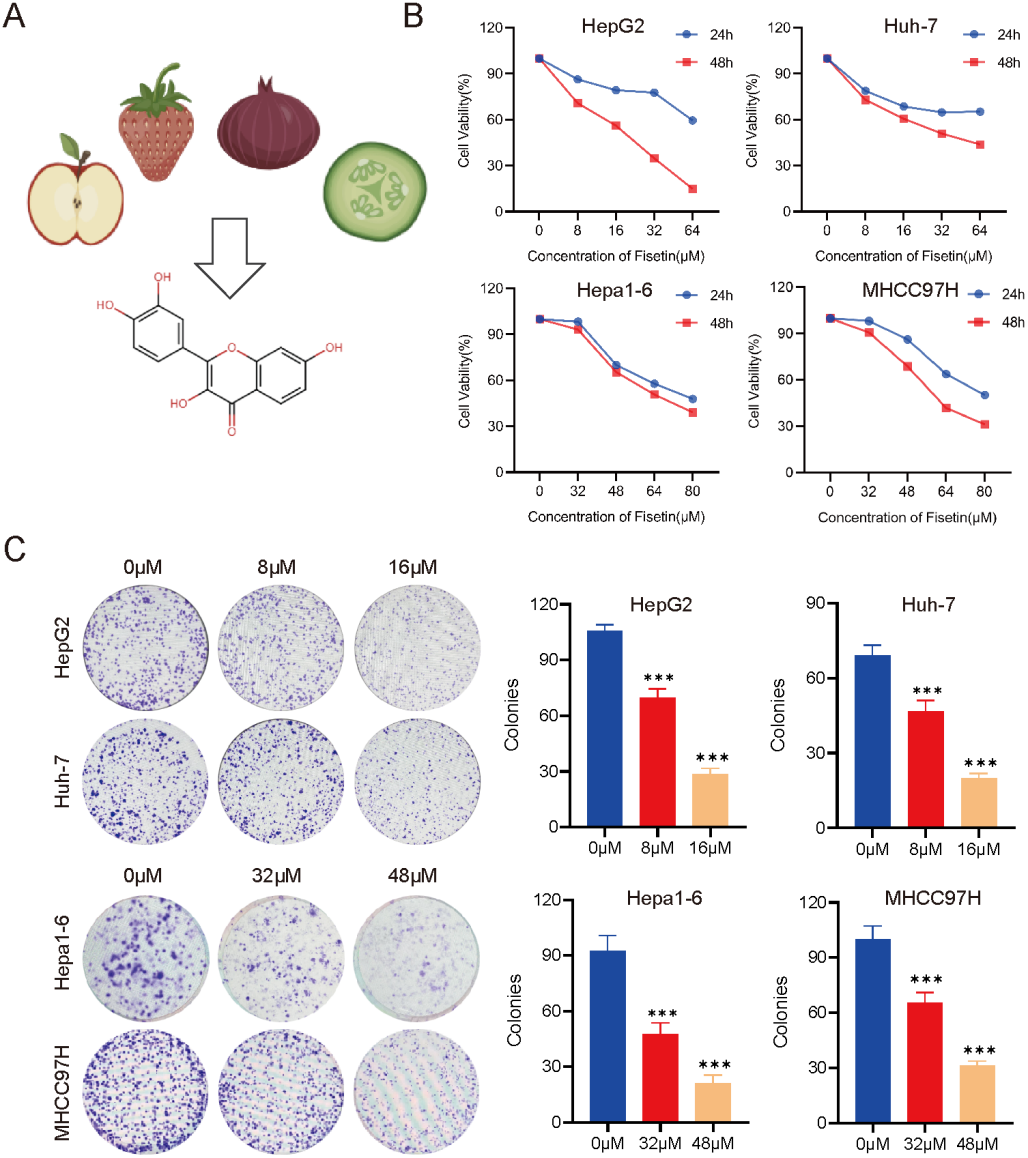
Fisetin inhibits the proliferation of liver cancer cells. (A) The chemical structure of fisetin. (B) CCK‐8 assay was used to detect the effects of different concentrations of fisetin on the proliferation of HepG2, Huh‐7, Hepa1‐6, and MHCC97H cells at 24 h and 48 h. (C) Colony formation assay was used to detect the effect of fisetin on the proliferation of liver cancer cells. ***p < 0.001.

The effect of fisetin on the proliferation of liver cancer cells was explored through CCK‐8 and colony formation assays. The CCK‐8 showed that the cell survival rate decreased after fisetin treatment. The IC_50_ of fisetin on HepG2, Huh‐7, Hepa1‐6, and MHCC97H cells at 48 h was 18.18 μM, 37.61 μM, 65.45 μM, and 60.58 μM (Figure 1B). The results of the colony formation assays showed that fisetin could inhibit the proliferation ability of liver cancer cells (Figure 1C). The above results indicated that fisetin could inhibit the proliferation of liver cancer cells.

### 3.2 Fisetin Inhibits the Migration and Invasion of Liver Cancer Cells In Vitro

**FIGURE 2:**
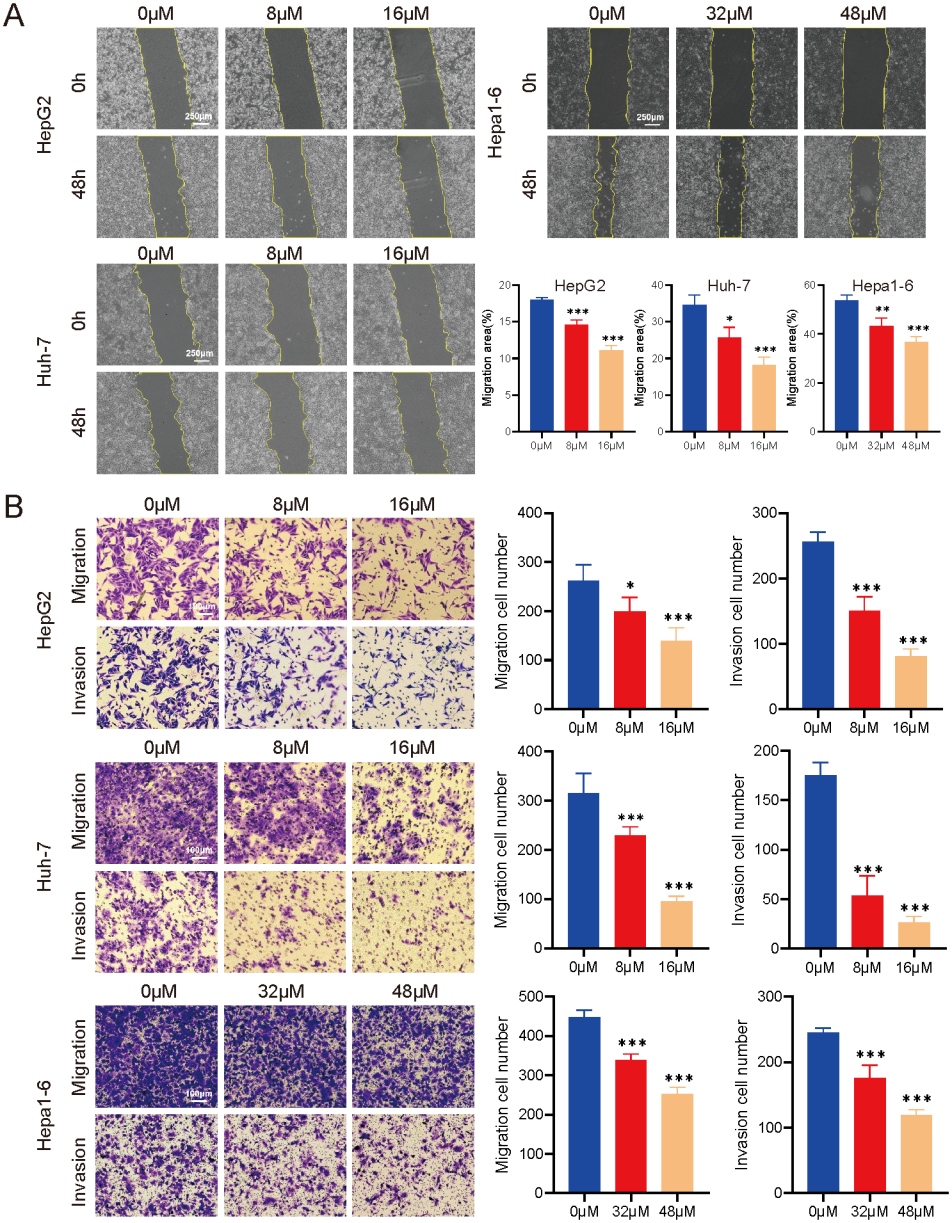
Fisetin inhibits the migration and invasion of liver cancer cells. (A) Wound healing assay was used to detect the effect of fisetin on the migration of HepG2, Huh‐7, and Hepa1‐6 cells. (B) Transwell assay was used to detect the effect of fisetin on the migration and invasion of liver cancer cells. *p < 0.05, **p < 0.01, ***p < 0.001.

The migration and invasion abilities of liver cancer cells in relation to fisetin were explored via the wound healing and the transwell assay. The wound healing assay results indicated that, compared with the 0 μM group, the percentage of migration area of HepG2, Huh‐7, and Hepa1‐6 cells decreased after fisetin treatment (Figure 2A). The results of the transwell assay showed that, compared with the 0 μM group, the number of cells that underwent migration and invasion decreased after fisetin treatment (Figure 2B). The above results indicated that fisetin could inhibit the migration and invasion of liver cancer cells in vitro.

### 3.3 Explore the Mechanism of Fisetin Against LC

**FIGURE 3:**
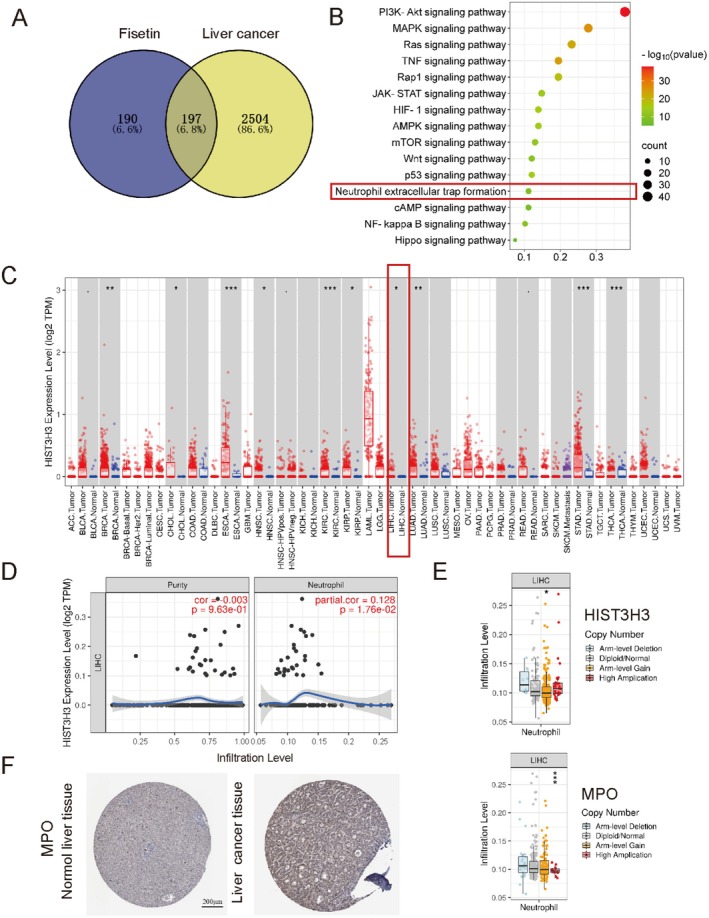
Explore the possible mechanism of fisetin against liver cancer by using bioinformatics. (A) The Venn diagram of the targets related to fisetin and liver cancer. (B) Bubble map of KEGG enrichment analysis of 197 genes. (C) The expression of HIST3H3 in different cancer tissues and adjacent normal tissues was explored using the TIMER database. (D) The relationship between HIST3H3 expression level and neutrophil infiltration level in liver cancer was explored using the TIMER database. (E) The correlation between copy number alterations in HIST3H3, MPO, and neutrophil infiltration was explored using the TIMER database. (F) HPA database was used to explore MPO expression levels in normal liver tissues and liver cancer tissues. *p < 0.05, ***p < 0.001.

Fisetin can inhibit the malignant biological characteristics of liver cancer cells in vitro. Therefore, we explored the mechanism of fisetin against liver cancer by using network pharmacology. A total of 387 fisetin‐related targets were collected by searching the TCMSP, SwissTargetPrediction, and Pharmmapper databases. A total of 2701 liver cancer‐related targets were obtained by searching the GeneCards and OMIM databases. One hundred ninety‐seven common targets were obtained (Figure 3A). The PPI network revealed core targets including TP53, TNF, AKT, and IL‐6 (Figure S1). The GO analysis indicated that hormone response, phosphorylation processes, and kinase activity were closely associated with the regulation of the tumor immune microenvironment. Additionally, cellular components such as membrane rafts and receptor complexes were implicated in immune signal transduction, suggesting that fisetin may exert its effects by modulating inflammatory responses and immune cell functions (Figure S2) KEGG analysis showed that there were a total of 108 related signaling pathways, including AMPK, mTOR, and the formation of NETs (Figure 3B).

In the tumor microenvironment, neutrophils recruited by tumor cells will release NETs under the stimulation of various cytokines (Zhang et al. 2024). Many animal models and clinical studies have shown that NETs play an important role in the occurrence and development of liver cancer. CitH3 is a recombinant protein of histone H3, and the high expression of HIST3H3 is closely associated with tumor progression. Through the TIMER database, we found that the level of HIST3H3 in liver cancer tissues was higher than that in normal tissues (Figure 3C). The level of HIST3H3 in patients with liver cancer was positively correlated with neutrophil infiltration (*p* < 0.05) (Figure 3D). Moreover, the changes in the copy numbers of HIST3H3 and MPO were significantly correlated with neutrophil infiltration (*p* < 0.05) (Figure 3E). By comparing the immunohistochemical results of MPO in the HPA database, it was found that the expression level of MPO in liver tumors was higher than that in normal liver tissues, indicating a higher degree of neutrophil infiltration (Figure 3F). Therefore, intervening in tumor‐related neutrophils and inhibiting the formation of NETs may become a new direction for the treatment of liver cancer.

### 3.4 Molecular Docking Verification

**FIGURE 4:**
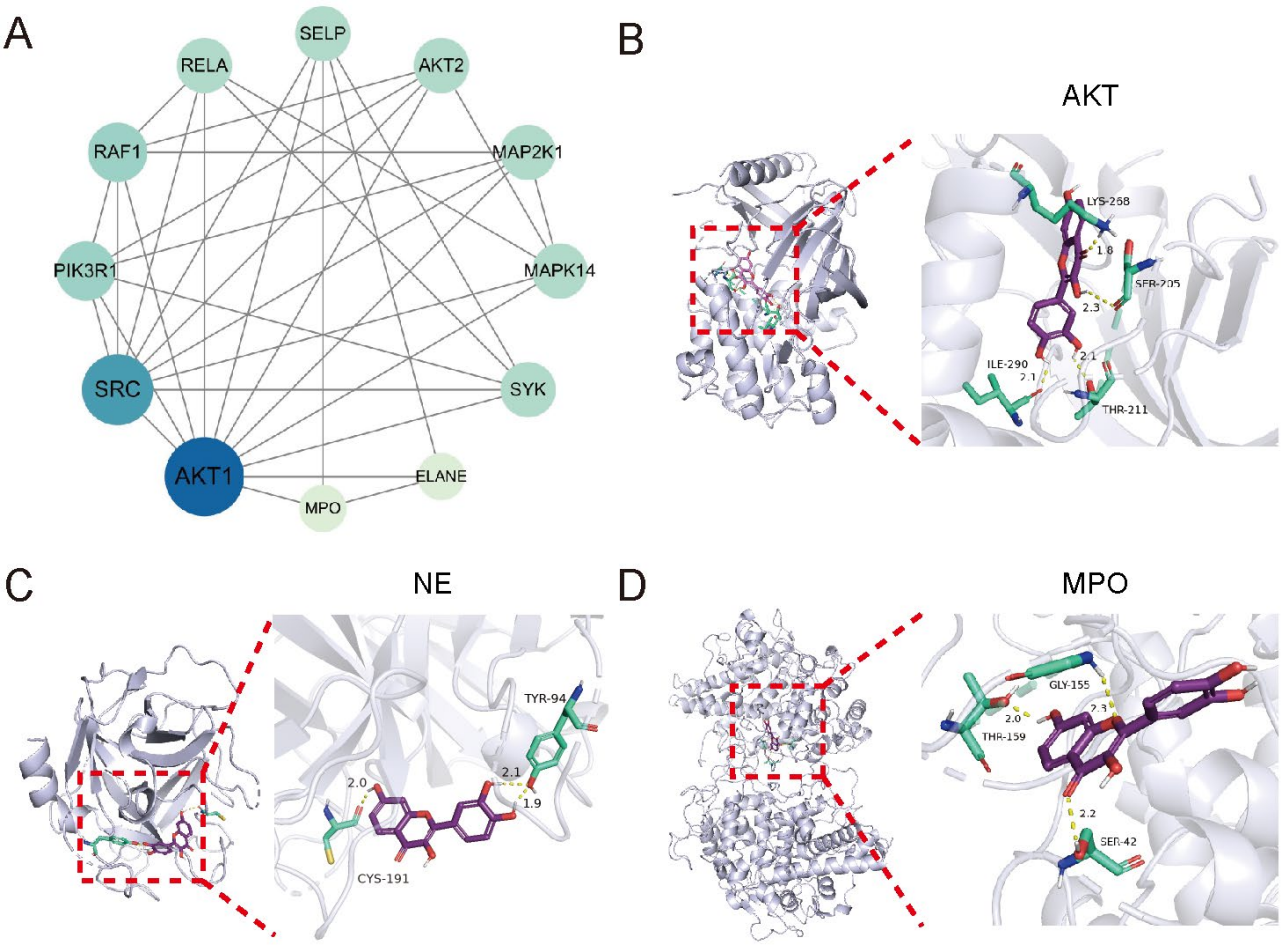
Molecular docking with NETs‐related targets. (A) Protein–protein interaction (PPI) network of NETosis‐related targets enriched in fisetin‐LC shared genes. Results of molecular docking of AKT (B), NE (C), and MPO (D) with fisetin.

We next sought to explore fisetin's potential interactions with NETs‐related proteins. The targets enriched in the NETs formation pathway are shown in Figure 4A. Notably, AKT serves as a crucial target both in liver cancer cells and during NETs formation. Current studies indicate that NET formation is a process dependent on ROS burst. Activation of AKT can directly enhance NADPH oxidase (NOX) activity, thereby promoting ROS generation and subsequently influencing NETs formation (Jiao et al. 2020). During NETosis, MPO and NE are released from azurophilic granules of neutrophils, where they participate in chromatin decondensation and NETs assembly. Molecular docking results demonstrated that fisetin binds to AKT, NE, and MPO with binding energies of −7.29, −7.24, and − 4.88 kcal/mol (Figure 4B–D). These results suggest fisetin may regulate NETosis, and the prediction was subsequently validated through experimental assays.

### 3.5 Fisetin Inhibits the Formation of PMA‐Induced NETs In Vitro

**FIGURE 5:**
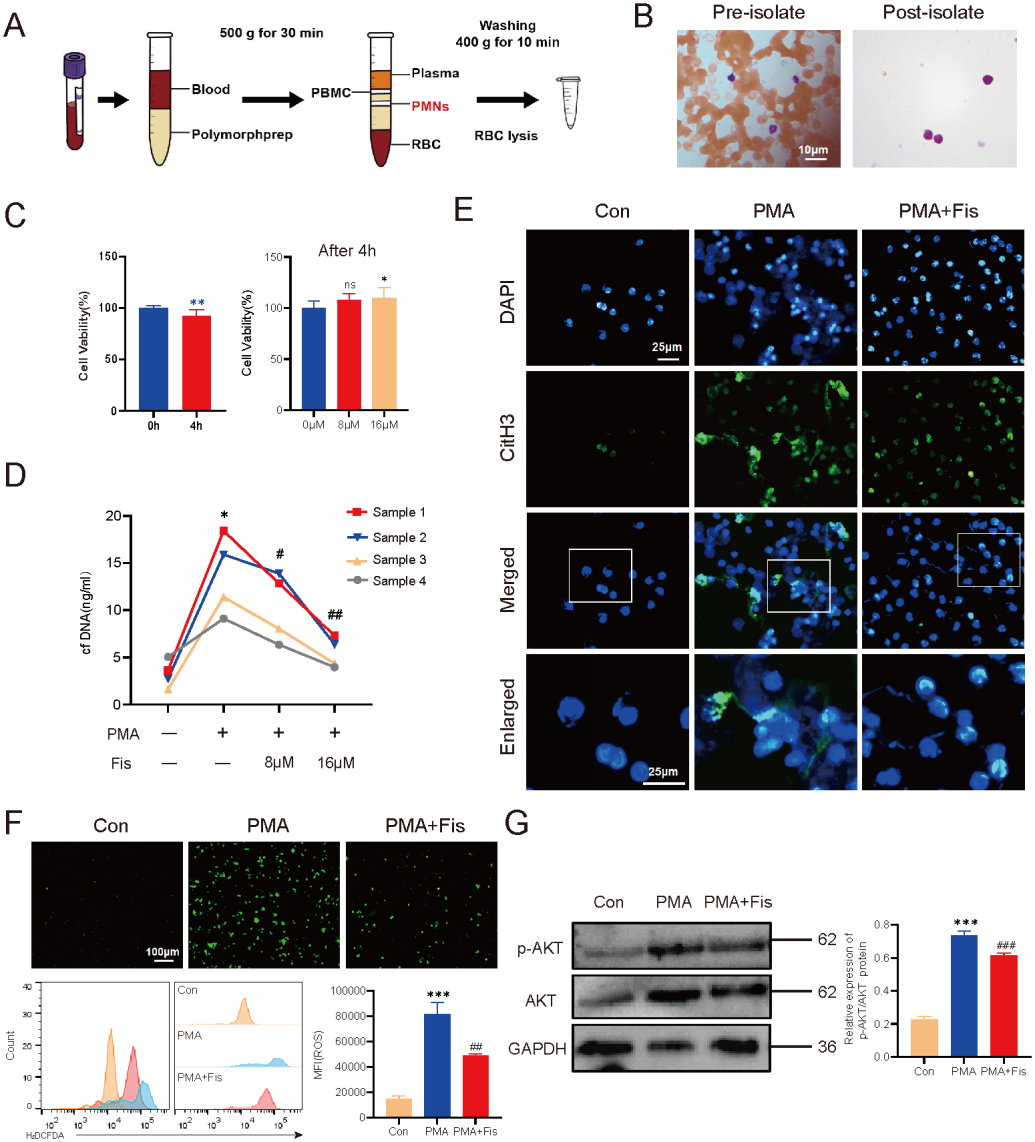
Fisetin inhibits the formation of NETs in vitro. (A) Schematic diagram of neutrophil isolation. (B) Neutrophil purity was determined by Swiss Giemsa staining. (C) The effect of culture time and fisetin on the viability of neutrophils was determined by CCK‐8 assay. ns *p* > 0.05, *p < 0.05. (D) PicoGreen dsDNA quantitative kit was used to detect cfDNA levels. **p < 0.01, vs. the first group. ##*p* < 0.01, ###*p* < 0.001, vs. the second group. (E) The level of CitH3 protein was detected by immunofluorescence assay. The concentration of fisetin in the PMA + Fis group was 16 μM. (F) The level of ROS in neutrophils was detected by using the fluorescent probe H2DCFDA. (G) WB was used to detect the protein levels of p‐AKT/AKT in neutrophils. ***p < 0.001, vs. the Con group. ##*p* < 0.01, ###*p* < 0.001, vs. the PMA group.

Bioinformatics analysis revealed elevated expression of NETs markers in liver cancer, while fisetin's core targets functionally overlap with NETs regulation. This dual evidence supports the hypothesis that fisetin's anti‐liver cancer action may partially rely on NETs suppression. Then, we collected EDTA‐anticoagulated whole blood from healthy volunteers and extracted neutrophils to explore the effect of fisetin on NETs in vitro. Firstly, the purity of neutrophil extraction was determined by Swiss Giemsa staining (> 95%) (Figure 5B), and the cell viability was detected by trypan blue staining (> 95%). The CCK‐8 results showed that the survival rate of neutrophils decreased significantly after 4 h of in vitro culture, while the survival rate of cells in the fisetin treatment group was higher than that in the untreated group. This indicates that 8 μM and 16 μM fisetin have no obvious cytotoxicity to neutrophils (Figure 5C). Subsequently, PMA was used to stimulate neutrophils to induce the production of NETs. The cfDNA level in the solution was quantitatively determined using the PicoGreen dsDNA kit, and the level of CitH3 protein in cells was detected by immunofluorescence. The results showed that the levels of cfDNA and CitH3 in the PMA group were significantly higher than those in the control group, indicating that NETs were successfully induced. However, after co‐treatment with fisetin and PMA, the levels of cfDNA and CitH3 decreased, indicating that NETs were inhibited (*p* < 0.05) (Figure 5D,E).

We used the fluorescent probe H2DCFDA to detect ROS in cells. The results showed that the MFI of cells in the PMA group was higher than that in the control group, while the MFI decreased after co‐treatment with fisetin and PMA. This indicates that fisetin can reduce the ROS induced by PMA (Figure 5F). The WB results showed that the protein level of p‐AKT/AKT in the PMA group was higher than that in the control group, while it decreased after co‐treatment with fisetin and PMA (Figure 5G), which aligns with our earlier molecular docking results. Therefore, it is speculated that fisetin may inhibit the formation of NETs by regulating the AKT/ROS axis in neutrophils.

### 3.6 Effects of NETs on Proliferation, Migration, and Invasion of Liver Cancer Cells

**FIGURE 6:**
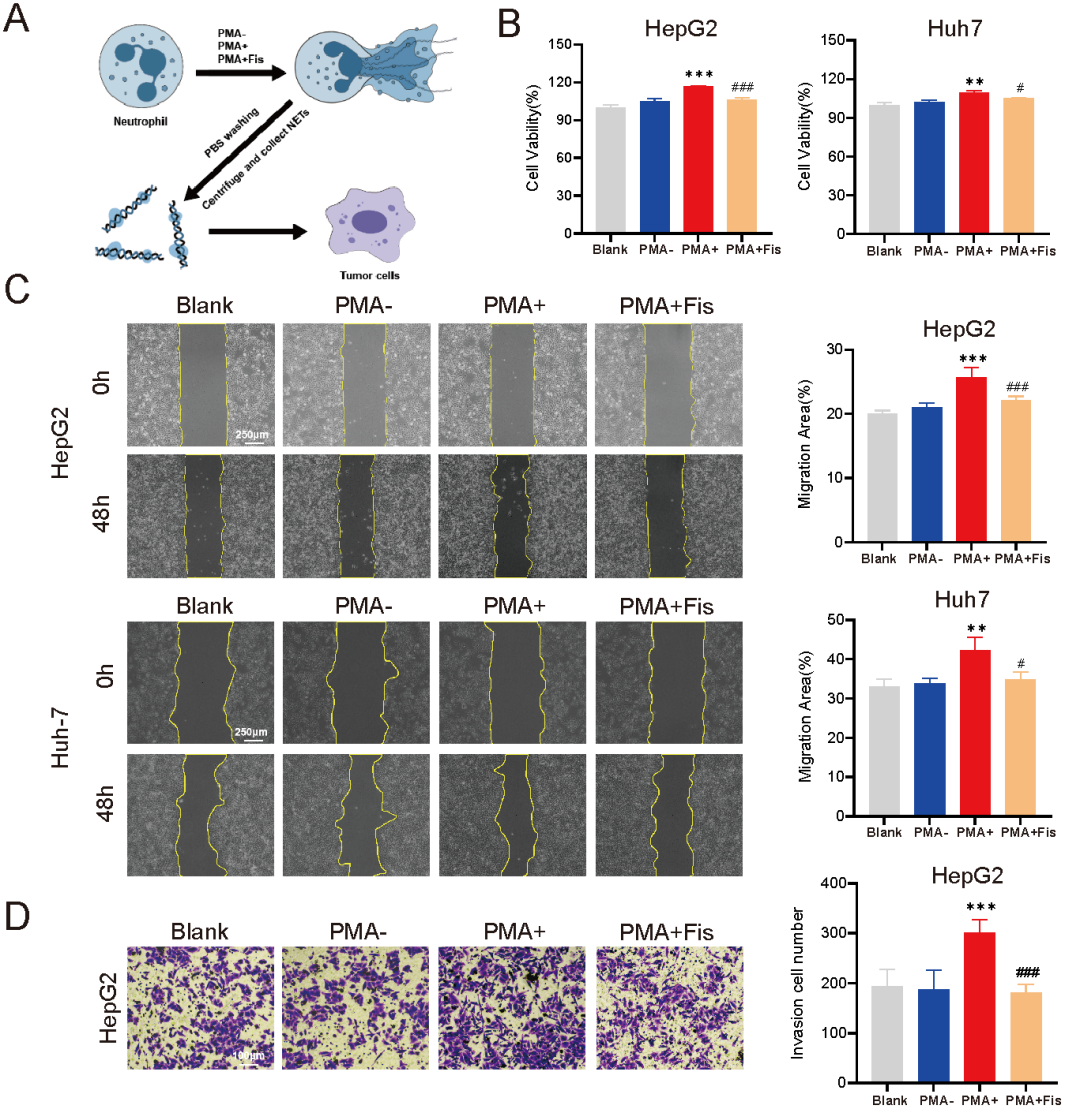
Fisetin attenuates the proliferation, migration, and invasion of liver cancer cells mediated by NETs. (A) Schematic diagram of the experimental process and grouping. (B) CCK‐8 assay was used to detect the effects on the proliferation of HepG2 and Huh‐7 cells. (C) Wound healing assay was used to detect the effect on the migration of HepG2 and Huh‐7 cells. (D) Transwell assay was used to detect the effect on the invasion of HepG2 cells. ** *p* < 0.01, ***p < 0.001, vs. the PMA‐ group. #*p* < 0.05, ###*p* < 0.001, vs. the PMA+ group.

To explore NETs' impact on liver cancer cells, we employed a conditioned medium‐based experimental approach, in which NETs components generated by neutrophils from different experimental groups were co‐cultured with HepG2 and Huh‐7 cells (Figure 6A). The blank group was added with the same volume of PBS. The results showed that the cell viability, migration area, and the number of invasive cells of liver cancer cells in the PMA+ group were all higher than those in the PMA‐ group, indicating that NETs could promote the proliferation, migration, and invasion of liver cancer cells (*p* < 0.05). However, the cell viability, migration, and invasion ability in the PMA + Fis group were lower than those in the PMA+ group (*p* < 0.05). This indicated that the formation of NETs decreased after neutrophils were treated with fisetin, and its effect on tumor cells was weakened (Figure 6B–D).

### 3.7 Fisetin Inhibits the Growth of Liver Cancer In Vivo and Suppresses NETs in Tumor Tissues

**FIGURE 7:**
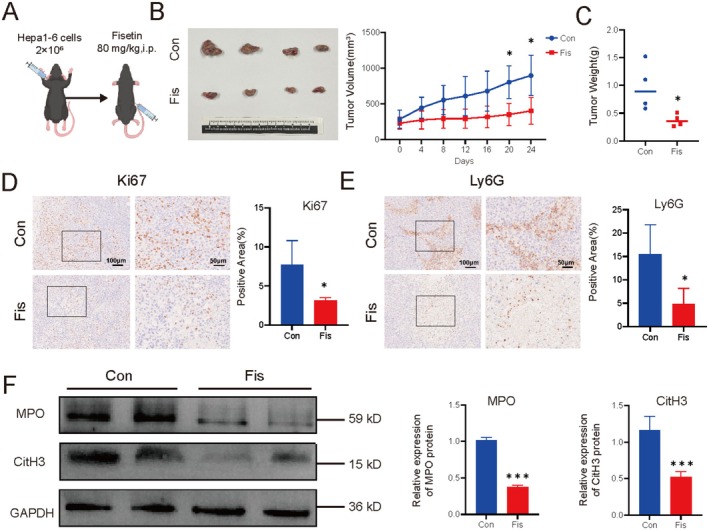
The role and mechanism of fisetin in anti‐liver cancer in vivo. (A) Schematic diagram of modeling and administration (*n* = 4). (B) Volume of subcutaneous tumors. (C) Weight of tumors. (D) Immunohistochemical detection of Ki67 protein expression in tumor tissues. (E) Immunohistochemical detection of Ly6G protein expression in tumor tissues. (F) The expression of MPO and CitH3 proteins in tumor tissues was detected by WB to evaluate the level of NETs. *p < 0.05, ***p < 0.001.

**FIGURE 8:**
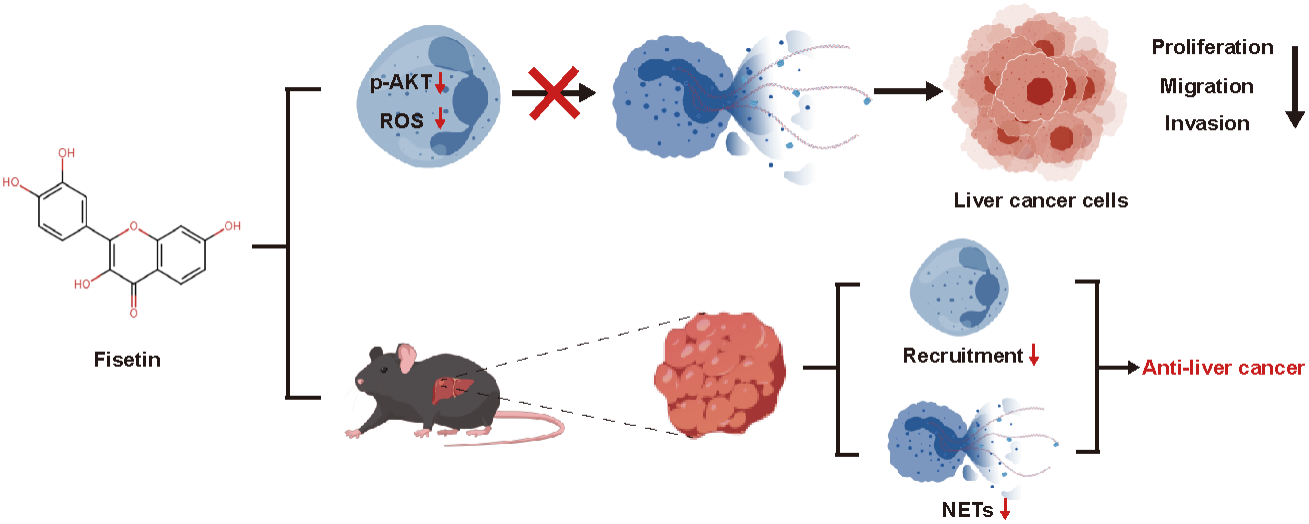
Schematic illustration of the effect and mechanism of Fisetin.

Subsequently, we established a subcutaneous liver cancer transplantation model in mice. The results indicated that the tumor weight and volume in the fisetin group were smaller compared to those in the control group (*p* < 0.05) (Figure 7B). The immunohistochemical results showed that the expression of Ki67 in the fisetin group was lower, indicating that fisetin could inhibit the growth of liver cancer (*p* < 0.05) (Figure 7C). After administration of fisetin, the expression of Ly6G in tumor tissues decreased, indicating that fisetin could reduce the infiltration degree of tumor neutrophils (*p* < 0.05) (Figure 7D). The WB results showed that the protein levels of MPO and CitH3 in the fisetin group were lower than those in the control group (*p* < 0.05) (Figure 7E). The above results indicated that fisetin exerted an anti‐liver cancer effect in vivo by inhibiting the recruitment of neutrophils and the formation of NETs (Figure 8).

## 4 Discussion

Current therapeutic strategies, including hepatic resection, liver transplantation, ablation therapy, transarterial chemoembolization (TACE), radiotherapy, and immunotherapy, have been widely employed in the clinical practice of liver cancer (Singal et al. 2024). However, a significant proportion of LC patients are diagnosed at intermediate or advanced stages, where surgical intervention is often no longer feasible. Moreover, radiotherapy and chemotherapy are frequently associated with severe adverse effects, and the development of drug resistance in tumor cells further compromises therapeutic efficacy (Cui et al. 2020). Consequently, the identification of novel therapeutic targets and the development of effective pharmacological agents for LC treatment are of paramount importance.

Many studies have shown that fisetin can inhibit the occurrence and development of various types of tumors, such as breast cancer (Talaat et al. 2023), pancreatic cancer (Jia et al. 2019) and lung cancer (Sabarwal et al. 2022). The mechanisms include inhibiting abnormal proliferation, malignant differentiation, and angiogenesis of tumors, promoting apoptosis and necrosis of tumor cells, inducing cell cycle arrest (Yang et al. 2024), reversing the occurrence of drug resistance (Zehra et al. 2024), etc. However, its role and mechanism in anti‐liver cancer remain to be further explored. We found in vitro, through CCK‐8, colony formation, wound healing, and transwell experiments, that fisetin can inhibit the proliferation, migration, and invasion of liver cancer cells. Then, bioinformatics analysis was used for further exploration and revealed that the mechanism might be associated with neutrophil extracellular trap formation.

Neutrophils are one of the most abundant cells in the immune system, accounting for the majority of white blood cells in the blood. In 2004, Brinkman pointed out that after being stimulated, neutrophils release a reticular structure composed of histones, DNA, and proteases, namely neutrophil extracellular traps (Brinkmann et al. 2004; Herre et al. 2023). The formation of NETs is regulated by multiple molecules and signaling pathways. After neutrophils are stimulated by various substances such as bacteria, viruses, lipopolysaccharides, PMA, and cytokines, their signaling pathways such as Raf–MEK–ERK and PI3K/AKT are activated, resulting in an increase in intracellular ROS production. Subsequently, protein arginine deiminase 4 (PAD4) is activated, causing citrullination of histones, leading to chromatin unwinding and expansion from the nuclear region to the cytoplasm. At the same time, MPO and NE are released from the azurophilic granules in the cytoplasm, and the neutrophil cell membrane ruptures, forming a reticular structure of NETs.

More and more studies have found that various drug components such as ginsenoside I (Lu et al. 2024) and icariin (Mou et al. 2024) can inhibit NETs. However, how natural products regulate the key molecules and pathways in the formation pathway of NETs still deserves further exploration. The AKT protein is very important in the process of NETosis. PMA can upregulate the phosphorylation of the AKT protein and promote the accumulation of ROS in neutrophils to induce NETs (Douda et al. 2015; He et al. 2023). Interleukin‐8 (IL‐8) produced by tumors can also lead to the phosphorylation of PI3K and AKT proteins in neutrophils, further resulting in the production of ROS and the formation of NETs (Zha et al. 2020). We identified a strong binding affinity between fisetin and the AKT protein through molecular docking. While fisetin's regulation of AKT has been well documented in various cell models, its effects in neutrophils remain unreported (Sun et al. 2021; Xiao et al. 2021). This knowledge gap prompted us to design a series of in vitro experiments for validation. In this study, we demonstrated for the first time that fisetin can inhibit the formation of NETs. The mechanism might be associated with the inhibition of AKT protein phosphorylation and the decrease in ROS production in neutrophils.

NETs not only serve as a defense mechanism of the body but also play a significant role in the occurrence and development of tumors. Tumor cells can recruit neutrophils to the tumor microenvironment and promote the occurrence of NETosis by releasing cytokines such as G‐CSF and IL‐8. The resulting NETs can facilitate the occurrence and development of tumors through processes such as EMT (Masucci et al. 2020), angiogenesis (De Meo and Spicer 2021), and cancer‐related thrombosis (Wang et al. 2024). Previous studies have found that the levels of serum MPO‐DNA in patients with liver cancer and CitH3 in liver cancer tissues were significantly increased. Moreover, the levels of NETs in intrahepatic metastases and lung metastases were higher than those in primary liver cancer (Yang et al. 2020). Blocking the TLR4/9‐COX signaling pathway can eliminate the inflammatory response triggered by NETs, thereby eliminating the metastasis potential of HCC caused by them (Zhan et al. 2023). The combination of DNase Ⅰ, which directly disrupts NETs, and anti‐inflammatory drugs such as aspirin/hydroxychloroquine can also effectively reduce HCC metastasis in mouse models (Guan et al. 2021). The above studies indicate that interfering with tumor‐related neutrophils and inhibiting the formation of NETs may become a new direction for the treatment of liver cancer. However, there are no reports yet on whether natural compounds can exert anti‐liver cancer effects by inhibiting the formation of NETs. In vivo experiments, we also found that fisetin can reduce the recruitment of neutrophils in tumor tissues and inhibit NETs to exert anti‐liver cancer effects.

## 5 Conclusions

In this study, we found that fisetin can exert an anti‐liver cancer effect by inhibiting the recruitment of neutrophils and the formation of NETs in tumor tissues. We proposed a new NETs inhibitor and provided a new target for the treatment of liver cancer. However, this study also has certain limitations. We did not conduct in vivo reverse verification using neutrophil‐depleted mice or PAD4 knockout mice. Nor did we deeply explore the effect of fisetin on the interaction between tumor cells and neutrophils. Multiple models still need to be supplemented for further verification.

## Author Contributions

**Jiahui Gao:** conceptualization, Data curation, Formal analysis, Validation, Writing – original draft. **Yujia Song:** investigation, Resources, Validation, Visualization. **Zanxiang Luo:** data curation, Methodology, Resources, Software. **Zejie Su:** formal analysis, Investigation, Methodology, Software. **Chengshi Fu:** formal analysis, Investigation, Software. **Anran Gao:** formal analysis, Methodology, Software. **Jingxiu Zhao:** formal analysis, Methodology, Software. **Lie Liu:** validation, Visualization. **Xiangyun Teng:** software, Validation, Visualization. **Jianhua Xu:** conceptualization, Data curation, Funding acquisition, Investigation, Resources, Writing – review and editing.

## Ethics Statement

All experiments were conducted in accordance with the Helsinki Declaration. Experimental research on vertebrates or any regulated invertebrates has been performed in accordance with the Basel Declaration. The Ethic Committee number of Shunde Hospital of Guanezhou University of Chinese Medicine is KY‐2024174. The Ethic Committee number of the Institutional Animal Care and Use Committee of Guangdong Medical Laboratory Animal Center is B202410‐9.

## Conflicts of Interest

The authors declare no conflicts of interest.

## Supporting information


**Data S1**.

## Data Availability

Data will be made available on request.
