# Supplementary material for: Study on the Mechanism of Fisetin Exerting Anti‐Liver Cancer Effects by Regulating Neutrophil Extracellular Traps
Source: Food Sci Nutr. 2025 Jun 18;13(6):e70309. doi: 10.1002/fsn3.70309 (PMC12174963; doi:10.1002/fsn3.70309)
Supplement: Supplementary file 1 — Data S1. [file FSN3-13-e70309-s001.docx]

**Supplementry material**


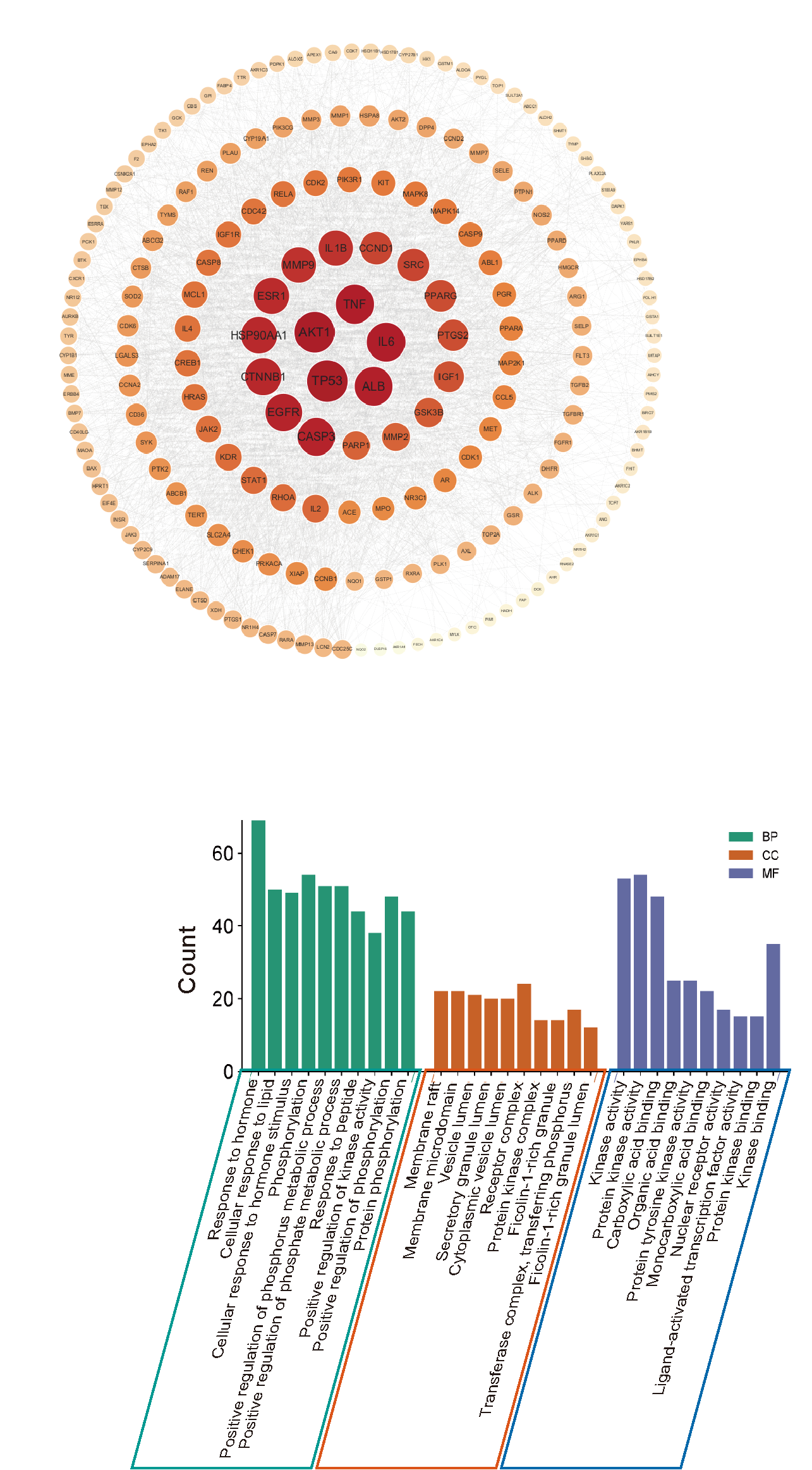


Fig. S1. The PPI network diagram of common targets of fisetin and liver cancer


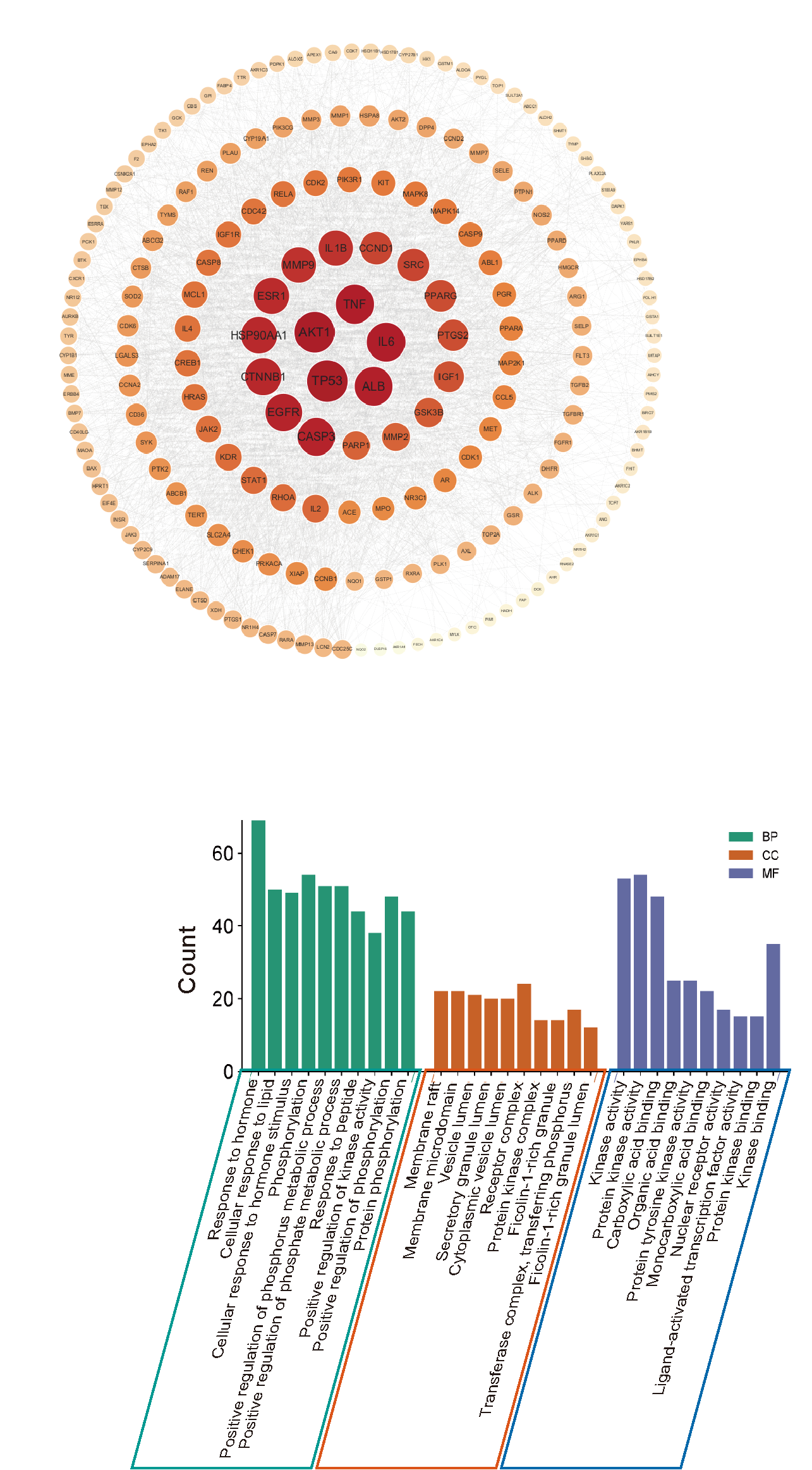


Fig. S2. GO enrichment analysis of 197 genes.
